# Benefit and Harm in the Early Management of Suspected Cervical Spine Injury: A Narrative Review

**DOI:** 10.7759/cureus.117067

**Published:** 2026-09-28

**Authors:** Omar T Al Zareeni, Abrar Azzabi, Anand Pillai

**Affiliations:** 1 Trauma and Orthopaedics, Stepping Hill Hospital, Manchester, GBR; 2 General Practice, Wrightington, Wigan and Leigh NHS Foundation Trust, Manchester, GBR; 3 Trauma and Orthopaedics, Wythenshawe Hospital, Manchester University NHS Foundation Trust, Manchester, GBR

**Keywords:** cervical collar, cervical spine injury, magnetic resonance imaging, spinal cord injury, spinal immobilisation

## Abstract

Suspected cervical spine injury is managed with a bundle applied largely by reflex: a rigid collar, sequential imaging, and, in some settings, corticosteroids, alongside interventions whose benefit depends on speed. This review sets the strength of evidence behind each component against the readiness with which it is applied. Sources were identified by targeted searching of PubMed/Medical Literature Analysis and Retrieval System Online (MEDLINE) and by reference tracking; the review is not systematic. The interventions applied most reflexively are the least supported. Prehospital immobilisation has never been shown to reduce neurological deterioration, spinal cord injury, or mortality, and the only study to compare an immobilising with a non-immobilising system found less neurological disability in the patients who were not immobilised. Pooled data across 25 studies give a pressure ulcer prevalence of 7% with prolonged collar use, and collar application raises intracranial pressure by 4.43 mmHg. MRI after a normal CT finds abnormalities in 17% of patients and changes management in 4%, with heterogeneity above 90%, and a practice management guideline has already concluded that the collar may be removed in obtunded patients after a negative high-quality CT alone. Methylprednisolone produces no long-term motor recovery and increases gastrointestinal bleeding. The interventions that do alter outcome are those most easily lost to delay: decompression within 24 hours yields 4.0 additional motor points, and 2.57-fold odds of two-grade improvement in impairment grade; hypotension carries a 2.71-fold risk of adverse functional outcome, and specialist referral reduces pulmonary embolism from 5.4% to 0.7%. Three national and consensus bodies have already moved away from routine rigid immobilisation. Initial management would be improved less by adding investigation than by withdrawing interventions of unproven benefit and protecting those that are time-dependent.

## Introduction and background

The cervical spine carries the weight of the head, protects the cord and permits a range of motion greater than any other spinal segment, and it is this combination of mobility and neural proximity that makes injury at this level so consequential [1].

Cervical spine injury carries the highest early mortality of any spinal injury and is more likely than injury elsewhere in the spine to produce spinal cord injury, usually as one component of polytrauma [2,3]. Traumatic spinal cord injury is uncommon but disproportionately costly. In a population-based study of 1,303 incident cases drawn from 4.9 million people, the age- and sex-standardised incidence was 26.5 per million per year, the mean age at injury was 59.2 years, 68.3% of patients were male, and 52.1% of lesions were cervical, with traffic collisions and occupational accidents each accounting for approximately 30% [4]. Mortality was highest for cervical lesions and rose steeply with age, the adjusted hazard of death within one month being ninefold greater in patients aged 75 years or more than in those under 55 [4]. The demographic is also shifting, with an increasing proportion of those affected being older adults injured by low-energy falls rather than younger adults injured at speed [5].

Two populations are discussed throughout and should be distinguished. Most patients managed for suspected cervical spine injury have no injury and no neurological deficit; they are exposed to immobilisation and imaging on the basis of mechanism alone. A much smaller group has confirmed spinal cord injury, in whom perfusion, decompression and specialist care apply. Interventions that are low value in the first group may be essential in the second, and the evidence below is presented with that distinction in mind.

The urgency of early management derives from the underlying biology. The initial mechanical trauma permeabilises neurones and glia and initiates a secondary injury cascade of ischaemia, oedema, excitotoxicity and inflammation that produces progressive cell death over the subsequent days and weeks [5]. The distinction between primary injury, fixed at the moment of impact, and secondary injury, which remains modifiable, is what makes the first hours worth studying at all: haemodynamic support, timely decompression and specialist care each act on the second process and none on the first [5].

The interventions applied earliest have a different provenance. Rigid immobilisation entered routine trauma practice by extrapolation from injury mechanics rather than through trial, and more than two decades ago it was argued that the policy subjected most patients to expensive, painful and potentially harmful treatment for little demonstrable benefit [6]. Comparable reasoning underpinned sequential imaging and, until the evidence turned against it, corticosteroid administration. These components reach almost every patient with a suggestive mechanism, and they reach them first.

Decisions about immobilisation, imaging, physiological support and referral are therefore made under time pressure and before a diagnosis exists. Delay or omission at any of these points has been linked to neurological deterioration, longer admission and higher mortality [7,8]. These decisions are not, however, equally well supported. Some parts of the standard bundle rest on mechanical reasoning rather than outcome data. Others have measurable, time-dependent effects on recovery and are constrained by how quickly a system moves rather than by any clinical uncertainty.

This review sets one against the other, examining for each component of early management how strong the evidence of benefit is, what harm has been quantified, and how readily the intervention is applied in practice. Two reviews of the initial management of cervical spine injury have appeared in this journal within the past eighteen months, both describing the standard pathway from prehospital care to definitive management [9,10]. This review takes a different approach. Rather than describing what is done, it sets the strength of evidence behind each component against the readiness with which it is applied.

## Review

Approach to the literature

Relevant literature was identified by targeted searching of PubMed/Medical Literature Analysis and Retrieval System Online (MEDLINE) using terms relating to cervical spine injury, spinal immobilisation, clinical decision rules, spinal cord injury and surgical timing, without date restriction and with no language limit applied at the search stage; the search was last updated in September 2026. This was supplemented by reference tracking of major trials, systematic reviews and guidelines. Studies were selected by the authors, with systematic reviews and meta-analyses preferred where one was available for a given clinical question, followed by large multicentre cohorts, randomised trials and guideline or consensus documents. Where more than one synthesis addressed the same question, the most recent or most comprehensive was used, and primary studies were cited where they supplied estimates not available from a synthesis. All cited studies were published in English. The searching was iterative rather than protocol-driven, and no single search string reproduces the reference list. No protocol was registered, the search was not exhaustive, and no formal appraisal of risk of bias or certainty was undertaken. The review is therefore interpretive and is presented as a framework for prioritisation rather than as a guideline.

Who is at risk?

In 250,584 major trauma patients, cervical spine injury was independently predicted by age of 35 years or more, a Glasgow Coma Scale below 15 (OR 1.35 to 1.85), systolic pressure below 110 mmHg (OR 1.16, 95% CI 1.02 to 1.31), road traffic collision (OR 2.58, 95% CI 2.26 to 2.94) and falls beyond two metres (OR 2.24, 95% CI 1.94 to 2.58) [2]. Head injury was not predictive.

Prevalence across 65 studies and 281,254 patients was 3.7%, rising to 7.7% in obtunded patients against 2.8% in those who were alert (p = 0.007) [3]. Assessability is therefore itself a risk factor, and clinical clearance is unavailable in precisely the group at highest risk. Suspicion should follow mechanism, physiology and the capacity of the patient to be examined, rather than reported symptoms.

Decision rules withhold imaging; they do not find injury

The two instruments in general use were built for one purpose. The Canadian C-spine rule was derived in 8,924 alert, stable patients with a Glasgow Coma Scale of 15, of whom 151 (1.7%) had a clinically important injury; on cross-validation it achieved 100% sensitivity (95% CI 98 to 100%) with 42.5% specificity, leaving a projected imaging rate of 58.2% [11]. The National Emergency X-Radiography Utilization Study (NEXUS) criteria were assessed in 34,069 patients with 818 injuries, achieving 99.0% sensitivity (95% CI 98.0 to 99.6%) and 12.9% specificity, and would have avoided imaging in 12.6% of those evaluated [12].

Pooled performance is consistent with the derivation data. Across 15 studies and 79,526 patients, the Canadian rule achieved sensitivity of 0.90 to 1.0 with false negatives of 0% to 0.11% and reduced imaging by 42%, while the NEXUS criteria achieved sensitivity of 0.83 to 1.0, false negatives of 1.0%, and reduced imaging by 30.9% [13]. Specificity was poor throughout, 0.01 to 0.77 and 0.13 to 0.46, respectively.

In the pooled data, the Canadian rule achieved higher sensitivity and a greater reduction in imaging than the NEXUS criteria, although both were evaluated in heterogeneous populations. The shared low specificity is the more useful observation. Both tools exist to withhold imaging safely in low-risk patients, and neither identifies injury. Neither can be applied to the obtunded, in whom prevalence is highest. A negative rule is permission not to image; it is not a negative diagnosis.

Imaging beyond the point of usefulness

Pooled sensitivity for cervical radiography is 52% (95% CI 47 to 56%) against 98% (95% CI 96 to 99%) for computed tomography [14]. Radiography misses about half of clinically significant injuries and has no role in clearing the cervical spine after high-risk blunt trauma.

MRI after a normal CT is a different problem. Across 11 studies and 1,535 patients, MRI found abnormalities in 16.6% after a normal CT; 4.9% were immobilised for longer as a result, and 0.7% had an injury requiring surgery found only on MRI [15]. Across 36 studies and 6,784 patients, injuries were found in 17% (95% CI 12 to 23%) and management changed in 4% (95% CI 2 to 7%), with heterogeneity above 90% [16].

Guidance has addressed this question directly. Applying the Grading of Recommendations Assessment, Development, and Evaluation (GRADE) framework to the obtunded adult blunt trauma patient, the Eastern Association for the Surgery of Trauma found no new neurological change after collar removal in 1,017 patients, and a best-case cumulative incidence of unstable injury of 0% across 11 studies and 1,718 subjects after a negative high-quality CT. It conditionally recommends collar removal after a negative high-quality CT alone [17].

Used routinely after a negative high-quality CT, MRI reliably finds something and rarely changes anything, and most of what it finds results only in continued immobilisation. This argument applies to routine use in the neurologically intact patient and not to MRI performed for indication. Where there are neurological signs suggesting cord injury, unexplained neurological deficit, or suspected ligamentous or cord pathology despite a normal CT, MRI remains appropriate and is recommended in national guidance. The distinction is between imaging driven by a clinical question and imaging performed because the CT was negative.

Immobilisation: measured harm, unmeasured benefit

Six studies of collars, blocks and spinal boards in prehospital and emergency care produced no clear evidence that immobilisation reduces neurological deterioration, spinal cord injury or mortality. The evidence was limited, heterogeneous and at significant risk of bias, and immobilisation increased pain and discomfort [18].

The nearest approach to a controlled comparison remains a retrospective study of two university hospitals with comparable physician training and resources, one of which immobilised all 334 patients transported from the injury site and one of which immobilised none of 120. After adjustment for age, sex, injury level and mechanism, there was less neurological disability in the patients who were not immobilised (OR 2.03, 95% CI 1.03 to 3.99, p = 0.04) [19]. A systematic review of eight observational studies found collar application in penetrating trauma associated with higher unadjusted odds of death (OR 8.82, 95% CI 1.09 to 194; and OR 2.06, 95% CI 1.35 to 3.13), with concealment of neck injuries and increased scene time [20]. That finding is specific to penetrating trauma, where the mechanism of a missed unstable injury differs fundamentally, and should not be extrapolated to blunt injury.

The harms in blunt trauma are quantified, and they are harms of prolonged immobilisation rather than of brief initial precaution. Applying a collar at the roadside for a short transfer and leaving one in place for days in an intensive care unit are different exposures, and the evidence below concerns the latter. Across 25 studies and 1,170 patients, prolonged collar use gave a pooled pressure ulcer prevalence of 7% (95% CI 4 to 13%), with increased dysphagia, mood disturbance and lower respiratory tract infection [21]. A separate systematic review found collar-related pressure ulcer incidence ranging from 6.8% to 38%, at the occiput, chin, shoulders and back, with severity from stage 1 to lesions requiring surgical debridement; time in the device, intensive care admission, injury severity and mechanical ventilation were the principal risk factors [22]. Collar application raises intracranial pressure by 4.43 mmHg (p < 0.01), and removal lowers it by 2.99 mmHg (p = 0.02) [23]. That matters most in the patient with concurrent head injury, who is also the patient who cannot be clinically cleared and will therefore remain in the collar longest.

Practice has begun to move. A critical review of prehospital collar use concluded that the evidence base is insufficient to support routine application and proposed an immobilisation strategy that excludes the collar [24]. A United Kingdom consensus statement from the Faculty of Pre-hospital Care reached a similar position [25]. A Danish national interdisciplinary guideline, built on a graded systematic review, issued a strong recommendation against spinal stabilisation in isolated penetrating trauma, a weak recommendation against prehospital rigid collar and backboard in physiologically stable patients, and a weak recommendation for the vacuum mattress in transport [26].

Few interventions in this pathway show so wide a gap between how often they are used and how well they are supported. These are observational comparisons and cannot establish that immobilisation causes harm, but the absence of demonstrated benefit alongside consistently measured harm argues for treating immobilisation as a provisional, time-limited measure requiring active reassessment rather than as a default state that persists until someone thinks to stop it.

Methylprednisolone

Across four randomised trials and 17 observational studies, methylprednisolone produced no improvement in long-term motor score (p = 0.55) and no increase in the proportion gaining at least one motor grade [27]. A 4.46-point short-term benefit appeared when the drug was given within eight hours (p = 0.01), in an analysis at high risk of bias and imprecision, alongside increased gastrointestinal bleeding (p = 0.02). A small, early, imprecise signal set against a quantified harm is not a basis for routine use.

What actually changes the outcome?

Perfusion and Oxygenation

Hypotension is associated with higher odds of an adverse functional outcome across 14 studies and 2,553 patients (unadjusted OR 2.71, p < 0.0001), for both mean arterial and systolic pressure [28]. The 2024 clinical practice guideline on haemodynamic management suggests augmenting mean arterial pressure to a lower limit of 75 to 80 mmHg without active augmentation beyond an upper limit of 90 to 95 mmHg, for three to seven days, while explicitly rating the underlying evidence as very low quality and both recommendations as weak [29]. Airway protection is therefore part of the same objective. Across 42 studies, none of 73 patients with spinal cord injury intubated by rapid sequence induction with manual in-line stabilisation deteriorated neurologically, supporting a level-B recommendation [30], and multi-society United Kingdom guidelines published in 2024 set out 23 recommendations for airway management in this population, noting that the supporting evidence is generally modest [31]. Where physiology and rigid immobilisation conflict, physiology takes precedence.

Decompression Within 24 Hours

Pooled individual patient data from 1,548 patients show 4.0 additional motor points after decompression within 24 hours (23.7 versus 19.7, p = 0.0006), with improvement in light touch (p = 0.0021), pin prick (p = 0.002) and impairment grade recovery at one year (p = 0.0019) [32]. A multicentre cohort found two-grade improvement in the American Spinal Injury Association Impairment Scale in 19.8% after early decompression against 8.8% after late decompression (OR 2.57, 95% CI 1.11 to 5.97) [7]. The 2024 clinical practice guideline recommends surgery within 24 hours of injury as the preferred option at any level, on moderate evidence that patients were approximately twice as likely to improve by two or more impairment grades at six months (RR 2.76, 95% CI 1.60 to 4.98) and at 12 months (RR 1.95, 95% CI 1.26 to 3.18), with an additional 4.50 motor points (95% CI 1.70 to 7.29) [33]. This is the strongest recommendation in the pathway and the only one supported by a guideline rated at moderate certainty. A baseline examination performed to international standards is what makes deterioration visible and urgency defensible [34].

Specialist Referral

Pulmonary embolism occurred in 0.7% after referral to a specialist centre against 5.4% where referral was delayed (p = 0.05), and pneumonia in 19.1% against 37.1% [8]. These are observational comparisons between differently organised services rather than evidence of a treatment effect, and centres differ in more than referral timing. The association is nonetheless consistent, and it is organisational rather than technical, which makes it the one most easily lost in a system under load.

The inversion

Table 1 sets the two groups side by side. Several of the interventions applied earliest and most universally have either no demonstrated benefit or a benefit confined to a minority of patients, and several carry measurable harm, although the certainty of the evidence differs considerably between them. By contrast, the interventions with the most consistent effects on neurological recovery are those most vulnerable to delay.

**Table 1:** Evidence of benefit and harm for interventions in suspected cervical spine injury AIS, American Spinal Injury Association Impairment Scale; CCR, Canadian C-spine rule; CT, computed tomography; ICP, intracranial pressure; MRI, magnetic resonance imaging; NEXUS, National Emergency X-Radiography Utilization Study; OR, odds ratio; SCI, spinal cord injury. Evidence in this table is drawn from references [7,8,11-23,27,28,32].

Intervention	Evidence of benefit	Evidence of harm
Prehospital rigid collar and board	No clear evidence of reduced neurological deterioration, SCI or mortality across six studies [18]; less neurological disability in the non-immobilised system in the only two-system comparison (OR 2.03, 95% CI 1.03-3.99) [19]	Pressure ulceration 7% (95% CI 4-13%) in 1,170 patients [21] and 6.8-38% across a second review [22]; ICP +4.43 mmHg [23]; dysphagia and lower respiratory tract infection [21]; increased mortality in penetrating trauma [20]
MRI after a normal CT	Management altered in 4% (95% CI 2-7%), heterogeneity > 90% [16]; surgical injury found only on MRI in 0.7% [15]	Prolonged immobilisation in 4.9% [15]; 0% cumulative incidence of unstable injury after a negative high-quality CT argues against routine use [17]
Methylprednisolone	No long-term motor recovery (p = 0.55) [27]	Gastrointestinal bleeding (p = 0.02) [27]
CT as first-line imaging	Sensitivity 98% versus 52% for radiography [14]	Radiation exposure; not quantified in the cited sources
Clinical decision rules	Sensitivity 100% (CCR) and 99.0% (NEXUS) in derivation; imaging reduced by 42% and 30.9% [11,12,13]	Specificity 0.01-0.77 and 0.13-0.46; not applicable to the obtunded [13]
Avoidance of hypotension	Adverse functional outcome OR 2.71 (p < 0.0001) in 2,553 patients [28]	Not applicable
Decompression within 24 hours	+4.0 motor points (p = 0.0006) [32]; two-grade AIS improvement OR 2.57 (95% CI 1.11-5.97) [7]	Not established
Specialist centre referral	Pulmonary embolism 0.7% versus 5.4% (p = 0.05); pneumonia 37.1% to 19.1% [8]	Not applicable

Four implications for practice follow, each subject to the limitations of the underlying evidence. Risk profiling by mechanism, physiology and assessability should determine who is imaged. A negative high-quality CT is sufficient to clear the cervical spine in most patients, and MRI is better used as an adjunct driven by specific indication than as a routine next step. Immobilisation is best treated as provisional and de-escalated as soon as the diagnosis allows, and the time-critical elements of care are those governing blood pressure, decompression and referral rather than collar application.

Limitations

This is a narrative review. The search was targeted rather than exhaustive, no protocol was registered, and no formal risk-of-bias or certainty assessment was applied, so the selection of evidence discussed is interpretive and a systematic review might weight it differently. Several of the underlying syntheses are themselves limited. The principal immobilisation review was constrained by few studies and significant risk of bias, the MRI meta-analysis carried heterogeneity above 90%, and the comparative immobilisation data are observational, with the two-system comparison retrospective and drawn from populations differing in more than immobilisation practice. Failure to demonstrate benefit is not the same as demonstration that an intervention cannot help, and several of the interventions discussed have simply not been tested in adequately powered studies. The argument concerns the balance of evidence across interventions rather than the certainty of any single estimate.

## Conclusions

Initial management of suspected cervical spine injury shows a recurring mismatch between how readily interventions are applied and how well they are supported, although the certainty of the evidence differs considerably between interventions. Prolonged rigid immobilisation, routine MRI after a negative high-quality CT and methylprednisolone are applied more readily than the available evidence supports, and each is associated with measurable harm. Maintenance of perfusion, decompression within 24 hours and prompt specialist referral are associated with more consistent effects on recovery, and are limited chiefly by how quickly a system can deliver them. Several national and consensus bodies have already moved away from routine rigid immobilisation. Outcomes may be improved more by reducing interventions of uncertain benefit, and protecting those that are time-dependent, than by adding further investigation.
